# A Minireview *on* Brain Models Simulating Geometrical, Physical, and Biochemical Properties of the Human Brain

**DOI:** 10.3389/fbioe.2022.818201

**Published:** 2022-03-28

**Authors:** Yassine Bouattour, Valérie Sautou, Rodayna Hmede, Youssef El Ouadhi, Dimitri Gouot, Philip Chennell, Yuri Lapusta, Frédéric Chapelle, Jean-Jacques Lemaire

**Affiliations:** ^1^ Université Clermont Auvergne, CHU Clermont Ferrand, Clermont Auvergne INP, CNRS, ICCF, F-63000, Clermont-Ferrand, France; ^2^ Universite Clermont Auvergne, CNRS, Clermont Auvergne INP, Institut Pascal, F-63000, Clermont-Ferrand, France; ^3^ Service de Neurochirurgie, CHU Clermont Ferrand, F-63000, Clermont-Ferrand, France

**Keywords:** brain surrogate, medical devices, mechanical properties, biocompatibility, leachables

## Abstract

There is a growing body of evidences that brain surrogates will be of great interest for researchers and physicians in the medical field. They are currently mainly used for education and training purposes or to verify the appropriate functionality of medical devices. Depending on the purpose, a variety of materials have been used with specific and accurate mechanical and biophysical properties, More recently they have been used to assess the biocompatibility of implantable devices, but they are still not validated to study the migration of leaching components from devices. This minireview shows the large diversity of approaches and uses of brain phantoms, which converge punctually. All these phantoms are complementary to numeric models, which benefit, reciprocally, of their respective advances. It also suggests avenues of research for the analysis of leaching components from implantable devices.

## Introduction

The human brain is a complex organ at both functional and structural levels, which is placed in a particular biomechanical environment, the intracranial space. In the world of materials aiming to simulate biophysical properties of the brain, the words model, phantom, and surrogate are often used indifferently (Reinertsen and Collins, 2006; Forte et al., 2018; Zhang et al., 2019) even if models and phantoms should rather be representations, whereas the true surrogate should substitute the brain. Realistically there is no true surrogate of the brain, and models and phantoms are in their infancy. Nevertheless, few and partial structures and functions of the brain can already be surrogated. Indeed restoration of brain, by repair and regeneration, can be feasible using biomaterials such as bioscaffolds (Modo and Badylak, 2019) and bioengineering of the environment of stem cells (Zimmermann and Schaffer, 2019). Brain computer interfaces working via neuronal signal analysis and/or activation of neuronal population or body segment or exoskeleton, can surrogate inefficient auto repairing or treatments and must deal with biomaterials (Jeong et al., 2020). The bright future of replacements and surrogates will have to face the complexity of interactions between multiple domains from materials to regulatory processes (Handa et al., 2020).

The physical simulation of the different dimensions of the brain is extremely challenging and there is no model, phantom or surrogate that simulates the function, the structure, the aspect and the biomechanics all at the same time. The types of materials and their assembling, in a more or less realistic way, are essentially determined by the uses, such as imaging and biomechanical studies, education, surgery, developments of medical devices (MDs), and assessment of numeric models. Thus the choice of materials is not dissociable from the purpose of the physical representation accounting the context of the applications and uses.

**FIGURE 1:**
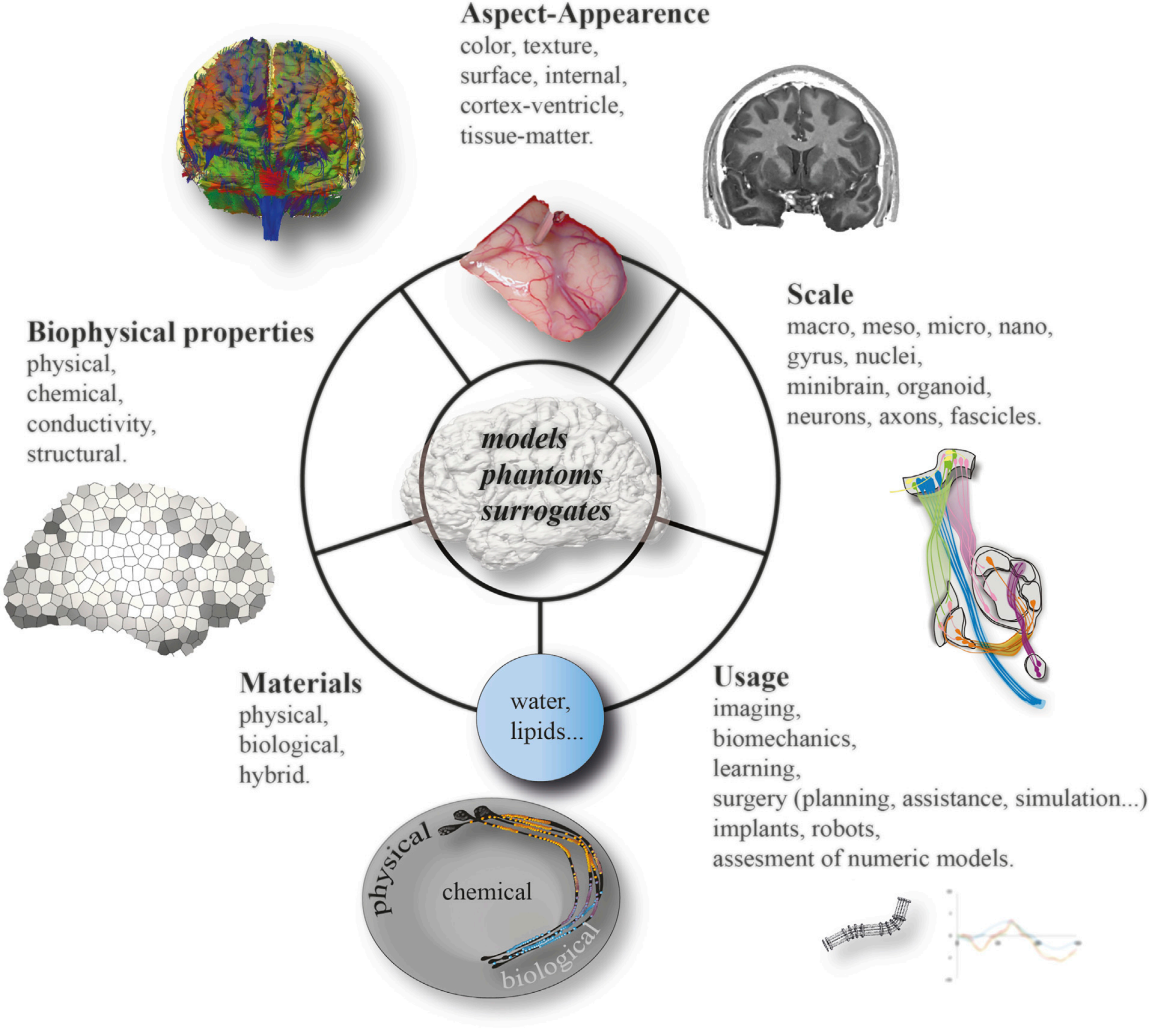
Overview of materials, models, phantoms and surrogates simulating the human brain.

Our goal was to carry out a mini-review of the materials proposed to simulate mechanical, chemical and biological properties of the human brain, as well as some of its structural elements such as architecture and aspect (Figure 1). The neural simulation of the brain function is particular because the functioning is so complex that it is still just not possible to simulate all the neuronal activity of the brain simultaneously, even with supercomputers. Recent programs illustrate the high level of the challenge (Amunts et al., 2016; Yamaura et al., 2020). Consequently, it was beyond our objective to integrate directly this dimension, in term of materials. The digital aspects of models are not addressed in this minireview. Hence we focused on the structural, physical and chemical properties of the brain, with the perspective of future medical applications, notably in neurosurgery, such as innovative treatments including surgery planning, as well as educational and training programs, which can be linked.

## Human Brain Models for Education, Training and Planning of Surgery

The realistic aspect of models, like their precise shape, size and colors, was largely skipped until recently. The most common human brain models for education are semi-realistic in the sense that they mostly aim to show the gross anatomy of the brain, in a more or less simplistic way (see e.g., search engine: “brain”+“model”+“education”), with some capabilities to see the “interior of the brain”, such as the ventricles. The targeted population of users is mainly undergraduate or graduate non-medical students. The models are generally made in rigid plastic (Azer and Azer, 2016) such as thermoplastic polyurethane (Goh et al., 2021), usually colored with different shades of pink, and specific colors highlighting particular regions, such as the hemispheres, functional territories or vessels. With the introduction of 3D printing, it has become easier to produce realistic “home-made” models, used for example, to explain diseases and therapeutic options to patients or relatives (van de Belt et al., 2018). Nevertheless, beyond the technological issues, the quality of data used for the 3D printing is variable. This quality is linked to the quality of medical images (geometrical and contrast resolutions, adequation between the type of image and the goal), the patience and diligence of the person in charge of the data extraction (as the best data is still extracted by skilled users), and the chain of data transfer from the raw data to the 3D printer. An advantage of additive manufacturing is that it enables the development of much more complex models, which could be able to integrate several physical dimensions of the brain (Zhao et al., 2020). The models used by neurosurgeons for training, preoperative planning and intraoperative guidance are promising (Rehder et al., 2016; Garcia et al., 2018; Qiu et al., 2018). However these models are still limited because the information embedded, such as topography, colors and texture, is not precise and they compete with virtual numeric models and historical anatomic dissections. For surgical training, physical models should add intracranial structures such as the vessels and the braincase (Ryan et al., 2016; Nagassa et al., 2019). One could expect that 3D bioprinting of physiologic or pathologic material, could be also used for training in surgery in line with the concept of mini-brain (Heinrich et al., 2019). However molding of synthetic materials can offer advantages such as low cost and easy making of brain surrogates, such as polymers and gelatins (Forte et al., 2018). More simple phantoms, made of radiopaque printed sheets intercalated with polyethylene foam layers, enable the design of anthropomorphic surrogates for training of interventional radiologists, with a fair CT-scan anatomic aspect, although they offer still limited haptic sensations (Jahnke et al., 2018).

## Human Brain Models for the Study of Medical Devices

The different materials used for the simulation of biophysical properties of the human brain aim to model at best one or more biophysical dimensions. The phantoms and models built from these materials depend on the usages, which are chiefly brain imaging analysis and study of mechanical stress. Specific models have been developed for special studies, such as agarose gel for intraparenchymal diffusion (Chen et al., 2004) or composite gel for dosimetry (Pavoni et al., 2015).

The phantoms used for experimental brain magnetic resonance imaging (MRI) or ultrasonic imaging, or those devoted to assessment of imaging, are essentially made of gels (Hellerbach et al., 2013). They enable the measurement of mechanical and thermic stress (Hellerbach et al., 2013; Sammartino et al., 2016), as well as MRI parameters such as diffusion and relaxation time (Fieremans and Lee, 2018), irrespectively of the architecture, at least the meso-architecture of gray nuclei, such as those of the thalamus and prethalamus, and of white matter (WM) tracts and fascicles, such as the cingulum and the brachium conjunctivum. Some agarose based phantoms allow the mimicking of metabolites during 7-T spectroscopic imaging, such as glutamic acid, creatine and phospho-creatine, myo-inositol, gamma-aminobutyric acid (GABA), choline chloride, sodium lactate and N-acetyl aspartate (Jona et al., 2021). Phantoms were also developed specifically for the neonatal brain (Kozana et al., 2018). The main limitation of these phantoms remains their non-realistic characteristics, notably structural, hence MRI brain models based on anatomic specimen are still relevant (Droby et al., 2015). Physical phantoms for ionizing imaging, CT-scan and Pet-Scan, are anterior and were designed for imaging and radiotherapy, notably in oncology. These phantoms can embed true bony or resin braincases. They are also able to simulate blood infusion (Boese et al., 2013) and they continue to be updated [e.g., (Mansor et al., 2017; Pourmorteza et al., 2017].

Additive manufacturing or 3D-printing, already enables to shape phantoms and to fill them with specific materials (liquid or solid) depending on the usages (Filippou and Tsoumpas, 2018). In the same line, the microarchitecture of WM fiber bundles could be embedded in the near future (Altermatt et al., 2019). In medicine, the measurement of mechanical stress distributed within the brain tissue enables the evaluation of the risks of lesioning and consequently of dysfunctions, although it is still challenging to infer functions from lesions. Future robotic and robotized surgeries will beneficiate from such data (Martin et al., 2009; Ruby et al., 2020). Besides the measurement of stress values, the determination of thresholds is pertinent as it enables the conception of protective solutions such as helmets, airbags and smart retractors. Phantoms were made of silicone (Margulies et al., 1990; Chanda et al., 2018; Zhang et al., 2019), gel (Reinertsen and Collins, 2006; Pomfret et al., 2013a; Awad et al., 2015) and dual material such as gel-polymer (Alley et al., 2011; Zhu et al., 2012). Agarose gels of 0.4–0.6% seem close to strain and rheology of bovine brain tissue (Pervin and Chen, 2011). Recent complex head models with a silicone rubber brain are used to study the dynamics of impact tests (Petrone et al., 2019). In parallel, the development of numeric models (Gabrieli et al., 2020) and atlases (Hiscox et al., 2020) continues to explore the complex biomechanics of the brain. It seems feasible in the near future to embed micro models of brain components, such as vascular tissue using silicone elastomer or hydrogel models (Sato and Sato, 2018), blood-brain barrier using hybrid silicone elastomer - plastic polycarbonate (Nguyen et al., 2019), up to mini-brains, organoids and brain-cell models using true human brain cells (Camp and Treutlein, 2017; Quadrato and Arlotta, 2017; Korhonen et al., 2018; Lovett et al., 2020). It is noticeable that most phantoms and models could be used to develop brain surrogates for education, training and surgery planning.

The simulation of electrical conductivity of the brain tissue is of upmost importance since the growing interest in invasive, such as the deep brain stimulation (Fariba and Gupta, 2020), and non-invasive, such as the transcranial magnetic stimulation (Lefaucheur, 2019), acute or chronic stimulations at frequencies usually below 200 Hz, of neurons and axons. Physical head phantoms have been developed to measure *in situ* computational models of electric fields, either caused by neurons or by external sources such as transcranial electric stimulation (Hunold et al., 2018; Magsood and Hadimani, 2021). Gel phantoms seem particularly interesting to study the electric conductivity (Kandadai et al., 2012; Pomfret et al., 2013b; Chew et al., 2014).

More recently, medical device biocompatibility, which relies on the ability of materials to perform with an appropriate host response in a specific application, gains increasingly in significance. At the tissue-material interface, two coupled aspects are present, the biotic factor that represents the cell and tissue reactions against the device, and the abiotic factor that represents the physico-chemical reactions at the surface of the material (Gulino et al., 2019). The study of biotic reaction relies on immortalized cells (Chapman et al., 2016; Mantione et al., 2016; Rejmontová et al., 2016; Koss et al., 2017; O’Rourke et al., 2017; Bradley et al., 2018; Johnson et al., 2018), organoids (Nasr et al., 2018; Nzou et al., 2018) and cultures (Persheyev et al., 2011; Mantione et al., 2016). Yet the study of the abiotic factor is still to be done, the related brain models being in the infancy, focusing on molecules and nanoparticles with animal protocols (Gulino et al., 2021; Ojeda-Hernández et al., 2021). The International Organization for Standardization (ISO) norm 10,993 evaluating the biocompatibility of medical devices, precises in part 18 (Chemical characterization of medical device materials within a risk management process, revised in May 2020) that an exhaustive investigation of extractible compounds must be performed and that the simulated extraction should be only performed when the total extractable components exceeds a tolerable limit. Anyway this approach could be insufficient to investigate the security of use of a medical device for two reasons: 1) exhaustive extractables need to be completed with a simulation performed in a physiological environment (Paskiet et al., 2013), and 2) because some leaching component are by nature endocrine disruptors (bisphenol A for instance) and could be more toxic in lower quantity than in high doses (Li et al., 2015).

## Discussion

Our minireview on the materials used to simulate mechanical, chemical and biological properties of the human brain, and structural features, shows that no model fulfills all these aspects. In parallel, the bio- mechanics and chemistry of the brain tissue should be present ideally in each brain models whatever the purpose. The biomechanical properties of the viscoelastic brain medium, is characterized by moduli, such as elastic and shear, and mechanical resonance. Recent MRI approaches, non-invasive, *in-vivo* and *ex-vivo*, yield more and more information, notably about the WM component such as the myelin density (Sepehrband et al., 2015), and about the WM anisotropy such as direction-dependent moduli (Smith et al., 2020). More specifically magnetic resonance elastography (MRE) enables the access to a large variety of physical parameters of the brain (Yin et al., 2018), notably the comparison of ex vivo and in vivo measurements of brain tissue (Chen et al., 2021) that enables to access to frequency-dependent behavior (Lv et al., 2020; Qiu et al., 2021)**.** Interestingly, MRE fast analysis of regional variations of biomechanics could measure variations of neuronal activity as shown in rodent model (Patz et al., 2019). Whatever the efforts done, there are still limited, robust, consensual values of physical parameters of human brain specimen, although the non-linearity of mechanical responses and the region dependency of behavior seems demonstrated (Budday et al., 2017). Data from animal have been harvested, such as the stiffness modulus of WM 1.895 ± 0.592 kPa, and of GM 1.389 ± 0.289 kPa of bovine (Budday et al., 2015). Nevertheless, although of interest, *ex vivo* data must be extrapolated carefully to *in vivo* human conditions (Karimi et al., 2019). On the other hand, the mass density is known, 1,046 ± 6, WM = 1,041 ± 2 and GM = 1,045 ± 8 [1,039–1,050] (Duck, 1990; McIntosh and Anderson, 2011). The main chemical components of the brain, water, lipids (O’Brien and Sampson, 1965; Dawson, 2015), other molecules such amino-acids and amides (Daković et al., 2013), and elements such as iron, copper and zinc (Grochowski et al., 2019), are well-known. The water content (g water/g tissue or %) ranges from 67 to 72 in WM and 80 to 87 in GM (Alexander and Looney, 1938; Whittall et al., 1997; Tofts, 2004; Oros-Peusquens et al., 2019). The proton density (percentage; water = 100) ranges from 69 to 77 in WM and 78 to 86 in GM (Tofts, 2004). Lipids’ concentration, pH, temperature, viscoelastic behavior and Young modulus are precised in the Table 1.

**TABLE 1:** Macroscopic chemical and physical properties of the human brain.

	Gray matter	White matter	References
Lipids concentration (in % of total weight)	7.00	16.02	(O’Brien and Sampson, 1965; Dawson, (2015)
Of which cholestérol concentration (%)	1.27	3.74	
pH	Between 6.8 and 7.2		(Xiong et al., 2004; Friese et al., 2007; Maddock et al., 2009; Orlowski et al., 2011; Magnotta et al. (2012)
Temperature	36.9 ± 0.4°C		Wang et al. (2014)
Viscoelastic behavior	Linear elastic		(Kaster et al., 2011; Budday et al., 2014, Budday et al., 2015, Budday et al. (2017)
Young modulus (kPa)	[1.038; 1.678]	[1.601; 2.487]	
Poisson ratio	0.45		


Lipid’s concentration depends on age and most are glycerophosphatides (i.e., ethanolamine glycerophosphatides, serine glycerophosphatides and choline glycerophosphatides) and cholesterol (O’Brien and Sampson, 1965; Dawson, 2015).

Concerning the medical device biocompatibility, the migration of compounds into a medium is described by the laws of Fick (Fick, 1855; Kaufmann, 1998) that estimate the transfer of material from an initial medium to a final medium accounting the contact area, gradient of concentration and diffusion coefficient. The temperature (Einstein, 1905; Demir and Ulutan, 2013; Wei et al., 2019), lipophilicity (Stein, 1981; Lodish et al., 2000; Brodeur and Tardif, 2005) and pH (Pinheiro et al., 1998) of the final medium influence the migration. In polymer, which are frequent in medical devices, the diffusion can deviate from predicted values, as a result of interactions between polymer and solvent slowing down the diffusion kinetics and the polymer gelation. Hydrogels, for example, characterized by the presence of water (or water-based solutions) in the polymer that enters or leaves the system can give rise to volumetric deformations. The transport of water in the glass phase is mainly driven by diffusion, which most of the time does not follow a pure “fickian” behavior (Caccavo et al., 2016). Another diffusion may occur, called abnormal diffusion (Goychuk, 2009; Yasuda et al., 2017), which is representative of viscoelastic diffusion (diffusion in relaxing media) that is affected by the mechanics of the system (Caccavo et al., 2018). The diffusion coefficient in a semi-solid and the viscoelastic properties of medium are correlated (Tanaka et al., 1973; Fujiyabu et al., 2019). For the brain viscoelastic medium with a linear elastic behavior, it is Young’s modulus E which is the most described. Its determination is made on the basis of connection with the shear modulus, by estimating that the Poisson’s ratio ʋ is equal to 0.45 (Paulsen et al., 1999; Clatz et al., 2003; Soza et al., 2004; Miga et al., 2016). To summarize, an adequate simulant for the study of leachables from medical devices must take into consideration the bicompartmental property due to the physicochemical difference between gray matter and WM, and must be prepared from components of high purity and meet the physicochemical characteristics.

In conclusion, future brain models should cover a wide field of applications in medicine, from those used for education, training and planning of surgery to those enabling the advanced study of medical device uses, notably their biocompatibility. Brain models, or phantoms, and digital brain models should learn from each other (Seo et al., 2022). It is anticipated that artificial surrogates will integrate most biomechanical and biochemical properties of the living tissue. Functional brain surrogates could be hybrid, made of nonbiological and biological components, and should communicate with the central nervous system for invasive prosthetic applications.
